# Silver Nanoparticles Addition in Poly(Methyl Methacrylate) Dental Matrix: Topographic and Antimycotic Studies

**DOI:** 10.3390/ijms20194691

**Published:** 2019-09-21

**Authors:** Valeria De Matteis, Mariafrancesca Cascione, Chiara Cristina Toma, Giovanni Albanese, Maria Luisa De Giorgi, Massimo Corsalini, Rosaria Rinaldi

**Affiliations:** 1 Department of Mathematics and Physics “Ennio De Giorgi”, University of Salento, 73100 Lecce (LE), Italy; chiara.toma@unisalento.it (C.C.T.); Marialuisa.degiorgi@unisalento.it (M.L.D.G.); Ross.rinaldi@unisalento.it (R.R.); 2 U.O.C. of Plastic Surgery and Burns Center, Department of Oral Hygiene Clini, Hospital “A. Perrino”, 72100 Brindisi (BR), Italy; giannialbanese69@libero.it; 3 Dental School, Interdisciplinary Department of Medicine, University of Bari “Aldo Moro”, 70124 Bari (Ba), Italy; massimo.corsalini@uniba.it

**Keywords:** silver nanoparticles, *Candida albicans*, poly(methyl methacrylate), dental prostheses

## Abstract

The widespread use of nanoparticles (NPs) in medical devices has opened a new scenario in the treatment and prevention of many diseases and infections owing to unique physico-chemical properties of NPs. In this way, silver nanoparticles (AgNPs) are known to have a strong antimicrobial activity, even at low concentrations, due to their ability to selectively destroy cellular membranes. In particular, in the field of dental medicine, the use of AgNPs in different kinds of dental prosthesis matrixes could be a fundamental tool in immunodepressed patients that suffer of different oral infections. *Candida albicans* (*C. albicans*), an opportunistic pathogenic yeast with high colonization ability, is one of the causative agents of oral cavity infection. In our work, we added monodispersed citrate-capping AgNPs with a size of 20 nm at two concentrations (3 wt% and 3.5 wt%) in poly(methyl methacrylate) (PMMA), the common resin used to develop dental prostheses. After AgNPs characterization, we evaluated the topographical modification of PMMA and PMMA with the addition of AgNPs by means of atomic force microscopy (AFM), showing the reduction of surface roughness. The *C. albicans* colonization on PMMA surfaces was assessed by the Miles and Misra technique as well as by scanning electron microscopy (SEM) at 24 h and 48 h with encouraging results on the reduction of yeast viability after AgNPs exposure.

## 1. Introduction

In the last century, several materials have been employed in dental and mandibular prosthetic reconstructions. Among the others, poly(methylmethacrylate) (PMMA) resin is widely used in medical and dental fields as a matrix in different kinds of removable or implantable devices due to its high biocompatibility, low cost, and ease of manufacture [1,2].

Nevertheless, clinical evidence has shown that dental prosthetic devices, mainly based on PMMA resins, undergo a proliferation of *C. albicans* infections [3,4,5], which affects their lifetime. Indeed, *C. albicans* is a pleomorphic *fungus* that is commensal of gastrointestinal microbiota [5] and, in case of immunodeficiency, the frequency of candidiasis can increase [6,7,8].

In order to eradicate *C. albicans* infections, several protocols for the periodic chemical cleaning of prostheses have been proposed; however these solutions are not definitive and, in addition, the treatment repetition causes damage to prosthesis surfaces, eventually compromising the longevity of implants [9,10,11,12,13].

Moving from these observations, structural modifications of PMMA matrices at nanoscale could be a possible strategy to improve their performance; in particular, resin implementation with NPs offers many advantages. Metal NPs, such as gold NPs (AuNPs) or titanium dioxide NPs (TiO_2_NPs), as well as mesoporous silica NPs (MSN), have been added to different kinds of biomaterials, such as PMMA-based matrices [14].

Lee et al. [15] employed MSN (2.5 wt%) as additive in PMMA obtaining a microbial reduction of 20–30% compared to the control samples, but this result needs to be improved.

Recently, Kim et al. [16] used carbon nanotubes (CNT) (0.25/2 wt%) as an additive in PMMA finding a reduction of *C. albicans*, *S. aureus,* and *S. mutans* adhesions of 35–95%. However, there are many concerns about the use of CNTs for clinical purposes [17]. AgNPs are the most used nanomaterial in different commercial products [18,19,20] and they are strong antibacterial [21] and antimycotic agents [22,23] as well as an anticancer tool [24,25] through their plasmonic features [26,27]. The exact mechanism of AgNPs toxicity is still unclear despite much evidence that has suggested it is their ability to release silver ions (Ag^+^) [28], stimulating molecular pathways that induce cell death [29]. Regarding the antimycotic properties, their activity against *C. albicans* has been demonstrated [30,31]. Panácek et al. [32] synthetized AgNPs by modified Tollens route, showing the antimycotic ability of NPs through an antiproliferation effect, even at low concentration (0.21 mg/mL). Similar results were obtained by Kim et al. [33]: AgNPs exhibited a strong inhibition activity on *C. albicans* that was greater than fluconazole, the drug usually used against the yeast. The antibacterial and antifungal activities of AgNPs support their employment in the biomedical field for prostheses and implants; in fact, the *C. albicans* infection is particularly aggressive on device surfaces due to their ability to develop biofilms on different materials (biological or inert) [34]. Roe et al. [35] showed the anti-growth ability of AgNPs on *C. albicans* in plastic catheters. Similar results were obtained by Hassan et al. [36], in comparison with the antifungal drugs grisofulvin, and itraconazole. In dental prostheses, AgNPs could reduce the oropharyngeal candidiasis which spreads in immune compromised individuals [37].

In our work we synthetized monodispersed citrate capped-AgNPs, having a mean size of 20 nm, and we added them in PMMA acrylic resin at two different concentrations (3 wt% and 3.5 wt%). 

The morphology of PMMA matrices enriched with AgNPs were characterized in terms of roughness by atomic force microscopy (AFM). Our results showed a strong reduction of roughness parameter, affecting the colonization and the proliferation of yeast, as confirmed by the Miles and Misra test and SEM analysis. 

## 2. Results and Discussion

The use of PMMA is widespread in dental and esthetic industries due to its low cost, but its surface porosity leads to several disadvantages [38]. Indeed, the dimensional instability of this material during the conventional heat-curing phase increases the number of pores in PMMA matrix becoming a suitable surface for microorganisms [39]. In addition, the hydrophobicity of PMMA contributes to increase *C. albicans* colonization [39,40]. In this work, we synthetized and characterized citrate capped-AgNPs, adding them to PMMA acrylic resin in order to improve its characteristics. 

**Figure 1:**
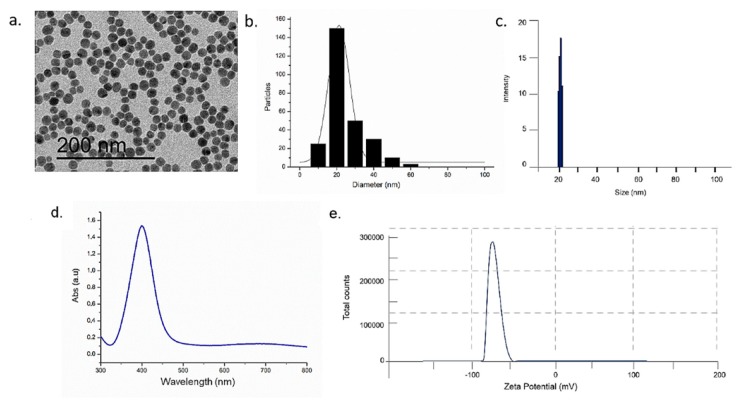
Characterizations of silver nanoparticles (AgNPs) in water: Representative TEM image (**a**), size distribution measured on 270 AgNPs and fitted with a normal function (solid line) (**b**), DLS (**c**), UV–vis (**d**) and ζ-potential measurement (**e**).

The morphology of AgNPs were characterized by TEM: They exhibited a spherical size of 20 ± 3 nm (Figure 1a), as showed on size distribution graph performed on 270 AgNPs (Figure 1b). The size of NPs was confirmed by DLS measurements: The hydrodynamic radius was compatible with the mean size values noticed in TEM acquisitions (19 ± 2) nm (Figure 1c).

UV measurements on AgNPs dispersed in ultrapure water showed the peak in UV region at ~400 nm of wavelength (Figure 1d). The surface charge was measured by ζ-potential measurements, showing the negative surface charge of NPs (−55 ± 2 mV), due to the citrate capping contribution (Figure 1e).

After NPs characterizations, we analyzed the morphology of PMMA-based resin, with and without AgNPs addition, at two concentrations (C1: 3 wt% and C2: 3.5 wt%). We retain that dental material architecture affects not only their performance, but also influences microbiological susceptibility.

In general, subjects wearing dental prostheses show an acidic and anaerobic oral environment, due to the adhesion between the oral mucosa and the prosthesis that is deprived of oxygen and saliva [41,42]. In addition, any small trauma on the mucosa caused by dental tools increases the permeability of *C. albicans* to invade the surrounding tissues [43]. In subjects suffering oral stomatitis and immunodeficiency, the *C. albicans* colonies produce acid proteinase and phospholipase on the surface prosthesis that promoted the adherence of the growing yeast, inducing several types of damage [44]. In this way, the Van der Waals and electrostatic forces between the yeast (that has a negative surface charge) and the surface play an important role [45]: Indeed, these forces promoted the adhesion that can be strong or less dependent on surface topography and chemistry [46]. Therefore, chemical modification of the surface charge of PMMA is important to prevent *C. albicans* adhesion. The addition of negative charged AgNPs reduced the roughness and yeast colonization on PMMA.

**Figure 2:**
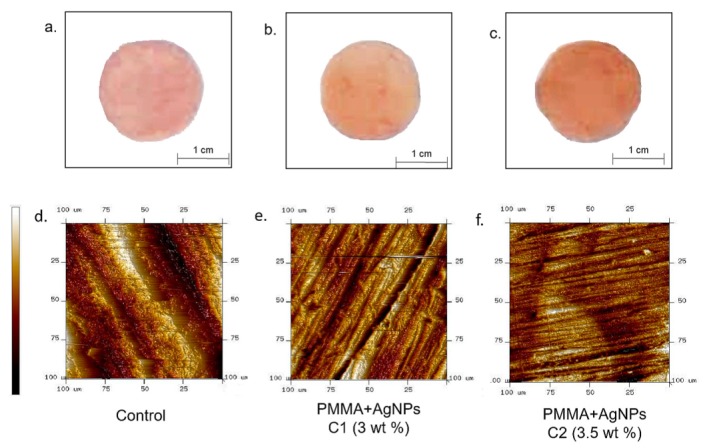
Images acquired by camera of poly(methyl methacrylate) (PMMA) without NPs added (control) (**a**); PMMA + AgNPs C1 (3 wt%) (**b**) and PMMA + AgNPs C2 (3.5 wt%) (**c**): The color became darker with increasing concentrations of the NPs. AFM topographical acquisitions of PMMA without NPs added (control) (**d**); PMMA + AgNPs C1 (3 wt%) (**e**) and PMMA + AgNPs C2 (3.5 wt%) (**f**).

At the two tested AgNPs concentrations (C1: 3 wt% and C2: 3.5 wt%), a little color change (from pink to dark pink/beige) of the PMMA resins, as consequence of AgNPs addition, was observed and compared to the untreated PMMA (Figure 2a–c). 

In order to quantify topographical differences of PMMA due to the presence of AgNPs, the surfaces roughness was measured by means of AFM. Topographic images (Figure 2d–f), clearly showed a flatter surface when AgNPs were implemented in PMMA in a dose dependent manner.

In fact, the PMMA surface was irregular and a lot of scratches and valleys were visualized (as indicated by a colorimetric scale associated to height of the sample); on the contrary, the presence of AgNPs in the matrix reduced sharp height alterations. 

**Figure 3:**
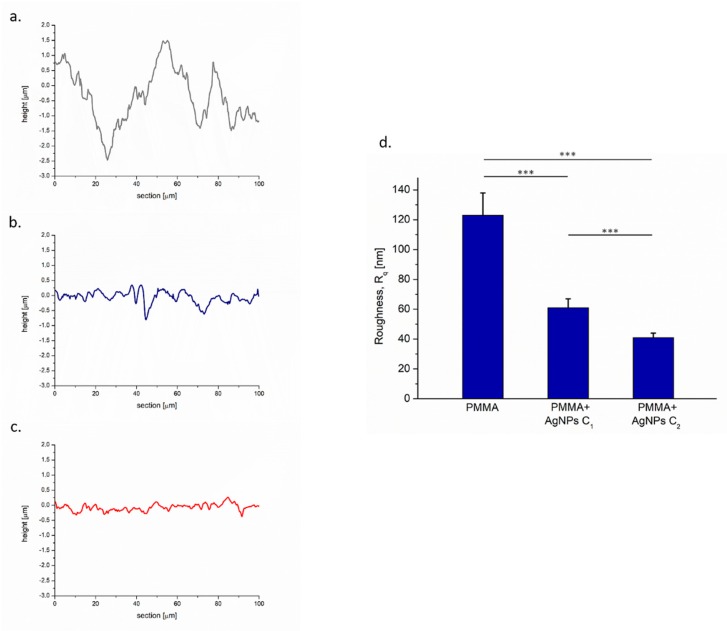
Representative cross-section profiles of PMMA surfaces without NPs added (**a**), PMMA + AgNPs C1 (3 wt%) (**b**) and PMMA + AgNPs C2 (3.5 wt%) (**c**). Roughness Rq values (nm) quantified on PMMA surfaces without NPs added (**a**), PMMA + AgNPs C1 (3 wt%) (**b**) and PMMA + AgNPs C2 (3.5 wt%) (**c**). (**d**) Data reported were calculated as average ± SD on three independent experiments, and the statistical significance respect to the control was represented (*** *p*-value < 0.005).

The topographical changes due to AgNP treatments were evident in cross-section profiles of PMMA surface (Figure 3a–c): Images showed flatter profiles after AgNP addition in dose dependent manner. This effect was quantified in terms of roughness (Rq) parameter.

These data were also confirmed from a quantitative point of view: Rq surface values of untreated PMMA changed from 123 ± 15 nm to 61 ± 6 nm and to 41 ± 3 nm, after AgNP addition at C1 and C2, respectively. Some experimental evidence reported how the ability of *C. albicans* to invade surfaces was related to roughness [47]; consequently, a smooth surface could decrease yeast colonization (Figure 3d).

**Figure 4:**
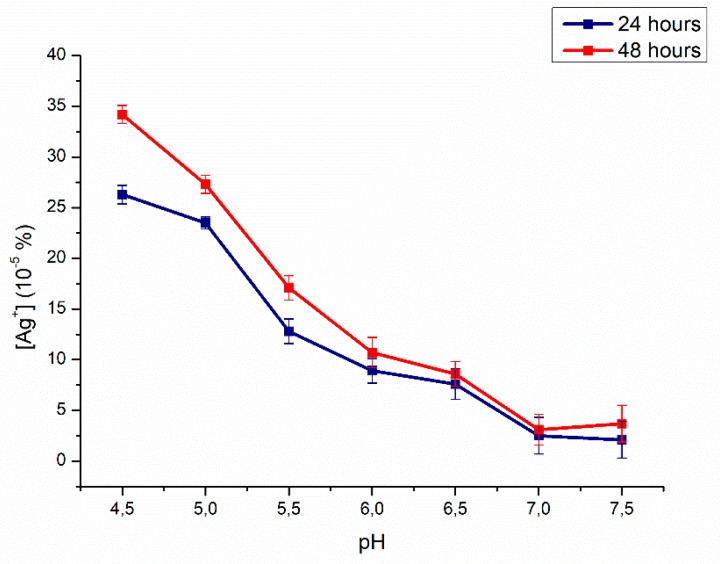
Effects of pH on Ag^+^ release from PMMA–AgNPs C2 (3.5 wt%). NP degradation was evaluated at pH 4.5, 5, 5.5, 6, 6.5, 7.5, and 7 up to 48 h.

Several investigators have shown that the radiation or the chemotherapy treatment in some kinds of cancers result in saliva pH change toward acidity and an increased incidence of *C. albicans* [48]. Some of these alterations have been recorded in patients with endstage renal disease [49] and diabetes [50]. In this environment, AgNPs have undergone a degradation process: The effect was particularly evident in acidic conditions [28]. For this reason, we tested the Ag^+^ degradation from PMMA–AgNPs C2 samples at seven pH points (from 4.5 to 7.5) up to 48 h. As showed in Figure 4, the effect was time and pH dependent: When pH values were lowered, the effect was stronger. For example, after 48 h, the Ag^+^ release was 35 × 10^−5^% for pH 4.5 in comparison with 0.48 × 10^−5^% at pH 7.5. However, the concentration of ions released from PMMA was very low and it was not considered toxic to human cells [28]. The disruption of *C. albicans* instead occurred even at slight Ag^+^ concentration [51,52,53]. 

When AgNPs were added to PMMA, these not only filled up the pores of the matrix, as reported by AFM analysis, but also probably made the PMMA surface electrically negative, due to their citrate capping. Moving from these observations, we speculated that AgNPs features could reduce the *C. albicans* colonization, so we carried out proliferation and adhesion tests.

**Figure 5:**
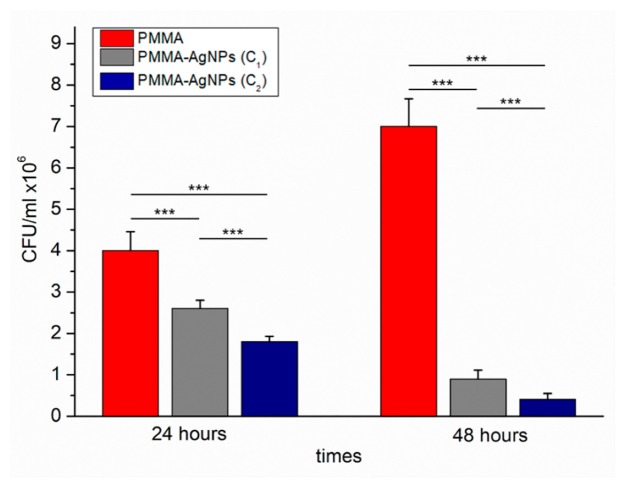
Viability of *C. albicans* on PMMA–AgNPs C1 (3 wt%) and PMMA–AgNPs C2 (3.5 wt%) were assessed by colony forming units (CFU) counts (the Miles and Misra test) after 24 h and 48 h of incubation. PMMA without AgNPs were also included as negative control. Data reported were calculated as average ± SD on three independent experiments, and the statistical significance respect to the control was represented (*** *p*-value < 0.005).

The Miles and Misra plate assay [54] was performed to assess the number of viable *C. albicans* CFU on PMMA, with and without the addition of AgNPs, at two tested concentrations (Figure 5). As reported, after 24 h of incubation, the CFU/mL were (2.6 × 10^6^ ± 0.6) for PMMA–Ag C1 (3 wt%) and (1.8 × 10^6^ ± 0.03) for PMMA–Ag C2 (3.5 wt%), respectively, whereas for the control PMMA we recorded (4 × 10^6^ ± 0.58) CFU/mL. 

Extending the time of incubation from 24 h to 48 h, the reduction of viability was more evident: when AgNPs were added in PMMA at higher concentration C2, CFU/mL was only (0.41 × 10^6^ ± 0.43), whereas on the PMMA without NPs (control) the CFU/mL was (7 × 10^6^ ± 1.03). Similar value was recorded for the blank: CFU/mL was (7.73 × 10^6^ ± 1.09), which referred to the CFU/mL of *C. albicans* grown in standard conditions for 48 h. Our data confirmed the antimicrobial properties of AgNPs against *C. albicans* in agreement with previous works [30,55]. 

**Figure 6:**
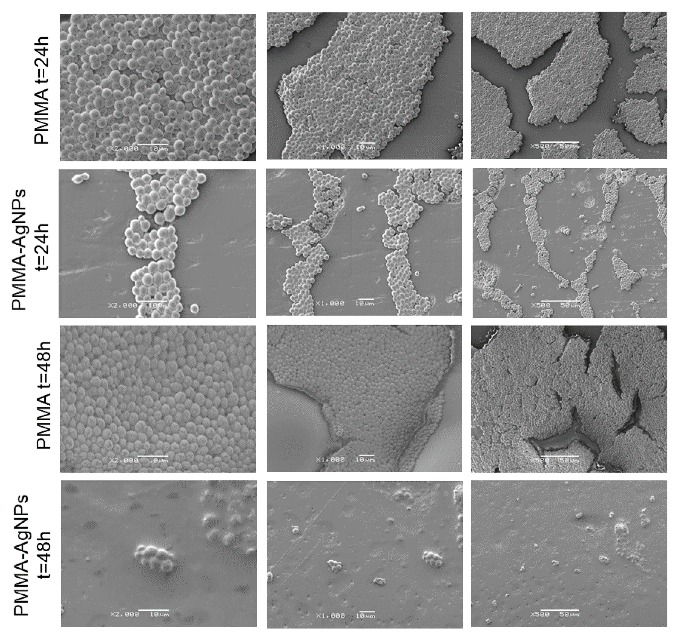
Representative SEM images at 3 magnifications (×2000, ×1000, ×500) of PMMA without NPs and after the addition of PMMA–AgNPs C2 (3.5 wt%) at different time points (24 h and 48 h).

We analyzed the ability of yeast to adhere on dental materials using PMMA–AgNPs C2 matrices and incubating them with *C. albicans* for 24 h 48 h. In details, at 48 h we assessed the surfaces of resins with and without AgNPs by SEM analysis (Figure 6). At time 0, little and isolated *C. albicans* colonies were found on the PMMA surface (data not shown), whereas, after 24 h, densely-packed yeast cells covering the entire surface were observed, using three different magnifications (2000×, 1000×, and 500×). At the same time point, the PMMA samples with AgNP addition showed a reduction of colonies that was evident after 48 h. We speculated that the reduction of colonization was mainly due to PMMA surface modifications exerted by AgNPs but also to AgNPs toxicity effects against microorganisms. 

The SEM acquisitions were used to assess the circularity of *C. albicans*. In general, the circularity parameter compares an object to a circle and it ranges from 0 to 1 (for a perfect circle) [56]. In our case, the loss of circularity corresponded to an increase of yeast mortality [57]. The morphology of control *C. albicans*, examined by SEM, exhibited homogeneous and regular cell surface both to 24 h and 48 h. Treated cells, in particular at 48 h, started to assume an abnormal appearance, reducing their circularity (Figure 6). This represented a first step that occurred at short times (24 and 48 h): in fact, at longer times the toxicity can be visualized due to the assumption of an irregular morphology, as demonstrated in previous studies [32,58,59]. 

**Figure 7:**
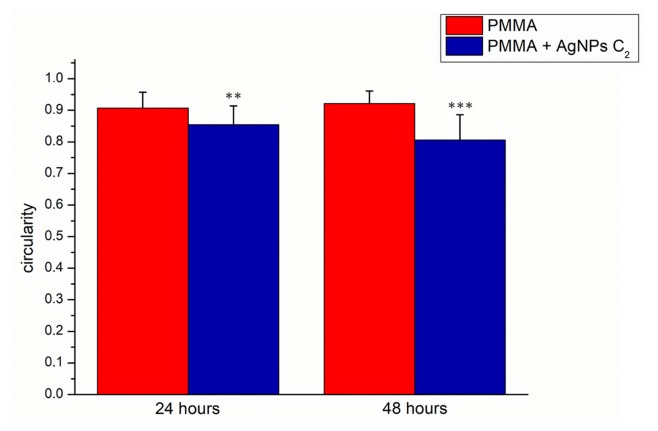
Histogram reported the mean values and their respective standard deviation of *C. albicans* circularity measured by ImageJ software on SEM acquisitions. The statistical significance of results respect to control cells was evaluated by *t*-test, and reported in histograms (** *p* < 0.01 and *** *p* < 0.005).

The circularity of *C. albicans* growth on PMMA was 0.91 ± 0.05 at 24 h and 0.92 ± 0.04 at 48 h. After the addition of AgNPs (C2), the circularity values were reduced from 0.85 ± 0.06 to 0.81 ± 0.08 at 24 and 48 h, respectively (Figure 7).

**Figure 8:**
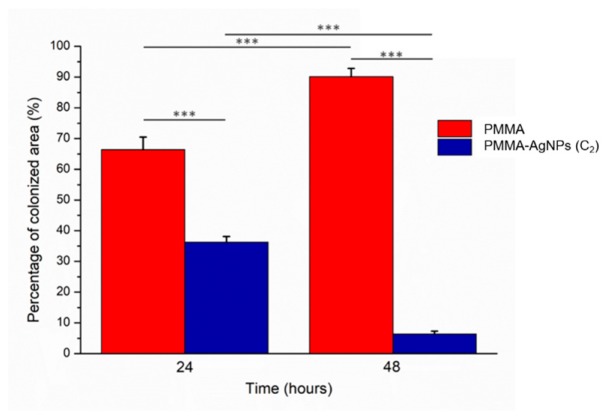
Histograms reported the colonization assay experiments of *C. albicans* on PMMA and PMMA–AgNPs C2 substrates. The analysis was conducted by ImageJ software on SEM acquisitions. The colonized area (**e**) was expressed as a percentage rate of the *C. albicans* covered area respect to entire acquired surface at two time points (24 and 48 h). Data reported were calculated as average ± SD on three independent experiments, and the values were considered statistically significant respect to control for *p*-value ˂ 0.05 (<0.05, <0.01 *, and <0.005 ***).

In order to establish the percentage of area covered by yeast on pure PMMA and PMMA–AgNPs C2, we used the software ImageJ (Figure 8). After 24 h of *C. albicans* incubation, the area covered by the microorganism was 66% ± 4% on the PMMA surface; it decreased to 36% ± 2% of coverage when the yeast was exposed to PMMA–AgNPs C2. After 48 h, the control colonization value 90% ± 3% was drastically reduced after AgNP addition, becoming 6% ± 1%. We speculated that the reduction of *C. albicans* adhesion on dental resins affected biofilm formation and influenced yeast viability [60,61].

The obtained results indicated that the implementation with AgNPs improved the performance of PMMA in prosthetic devices. In fact, we found that PMMA/AgNPs composite material had superficial structures that inhibited the colonization and viability of *C. albicans*. Currently, the development of *C. albicans* infections represents one of the main limitations to the duration of dental implants; for this reason, we strongly believe that the achievements of our work could have great impact in the biomedical and dentistry fields because they provide a preventative strategy for candidal biofilms formation on the denture surfaces.

## 3. Materials and Methods

### 3.1. Synthesis of AgNPs 

AgNPs were obtained following the route described in [28]. Briefly, AgNO_3_ (0.592 mM final concentration) aqueous solution was added to trisodium citrate (1.36 mM) and tannic acid (2.9 µM) and boiled at 120 °C. Then, the solution was cooled at room temperature and centrifuged at 6000× *g* for 45 min. AgNPs were collected and stored in the dark at 4 °C.

### 3.2. Transmission Electron Microscope (TEM), Dynamic Light Scattering (DLS), ζ-Potential, UV–vis Measurements (UV–Vis)

TEM images were recorded by a JEOL Jem 1011 microscope operating at an accelerating voltage of 100 kV. TEM samples were prepared by dropping a dilute solution of AgNPs in water on carbon-coated copper grids (Formvar/Carbon 300 Mesh Cu). Microscopy observations were made by means of a Scanning Electron Microscope (SEM, JEOL JSM-6480LV operating at an accelerating voltage of 10 kV, JEOL USA, Inc., Peabody, MA, USA). The sample was prepared by dropping a solution of AgNPs in water on monocrystalline silicon wafer. DLS and ζ-Potential measurements were performed on a Zetasizer Nano ZS90 (Malvern, Worcestershire, UK). Measurements were made at 25 °C in milliQ water and in cell culture medium used for cell experiments after 48 h of incubation. The optical absorbance spectra of AgNPs in water was measured with a Cary 300 UV–vis spectrophotometer (Varian, Palo Alto Palo Alto, California, USA) at a resolution of 1 nm using 5 mm path length quartz cuvettes.

### 3.3. Inductively Coupled Plasma Atomic Emission Spectrometer (ICP-AES) 

To quantify the metal content of NPs, after centrifuge in water, a little amount of solution was collected and digested by the addition of a solution of HNO_3_ 10% (*v*/*v*) over night. Afterwards, after dilution of ultrapure water, the amount of free ions was measured by ICP-AES (Varian Vista AX spectrometer, Palo Alto, California, USA).

The evaluation of AgNP ions release was performed at 37 °C at specific pH conditions ranged from 4.5 to 7.5. Acidic pH points (4.5, 5, 5.5, 6, 6.5) were prepared adding HCl to MilliQ water, whereas pH 7.5 was obtained adding NaOH. In each solution, three replicates of PMMA–AgNPs C2 were added. The ions release was analyzed at 24 h and 48 h. At each time points the solutions were collected and digested by the addition of of HNO_3_ 10% (*v*/*v*), and the number of free ions was measured by ICP-AES.

### 3.4. Synthesis of PMMA Resin/ AgNPs Composite Material

3 wt% and 3.5 wt% of AgNPs were added to 5 g of powder Resin Paladon 65 (Kulzer) and manually mixed using a steel spatula. After 3 min, the resin turned from a semi-liquid state to a semi-viscous phase and was pour slowly into silicon molds (80 shore). Then, the samples were inserted into a polymerization machine (Pentatlon 205, Effegi Brega, Sarmato, PC - Italy) for 30 min at 100 °C of temperature and 5 atm of pressure.

After polymerization, all the samples were treated after polymerization as follows:

- Stitch—with a multi-blade tungsten carbide bur mounted on a rotating instrument (50,000 rpm);
- Finishing—obtained in two-step: The first, using a thin abrasive cloth (75 microns) mounted on a spindle and, the second with a fine-grain finishing cutter (50 microns) mounted on a rotating instrument (50,000 rpm);
- Polishing—also obtained in two stages, the first, by using a cotton cloth brush mounted on a laboratory cleaner with water and pumice powder, and the second step with a dry cotton canvas and polishing liquid for resins (Dentaurum, Ispringen, Deutschland).

### 3.5. Atomic Force Microscopy (AFM) Analysis

The experiments were conducted by means of an advanced scanning probe microscope (Bioscope Catalyst, Bruker Inc., Santa Barbara, CA, USA), implemented on an inverted optical microscope (Zeiss Observer Z1, Zeiss, Jena, Germany). The whole system is placed on an insulating base to minimize effects of environmental mechanical vibrations on measurements. The experiments were performed using a V-shaped Bruker’s Sharp Microlever (MSNL, Bruker Inc., USA), that consists in high sensitivity Silicon Nitride cantilever. The topographic images were acquired on 50 × 50 µm scan area at resolution of 512*512. The images were analyzed by NanoScope Analysis software (Bruker Inc., USA) in order to quantify roughness parameters, and thus evaluate porosity at surface level. The roughness was expressed as Rq. In details, Rq was calculated as root mean square of height fluctuations respect to mean height value obtained by all data image, previously treated with a second order plane fit and with a second order flattening for deleting every bow and minimizing tridimensionality effects. To obtain a more accurate estimate of local roughness, Rq value was reckoned as the mean value of 20 selected areas of 3 × 3 um.

### 3.6. Microbiological Tests 

#### 3.6.1. *C. albicans* Culture Conditions

For the preparation of the *C. albicans* suspensions, the cells were inoculated in Sabouraud dextrose broth (SDB) (Oxoid, Hampshire, UK), incubated for 18 h at 37 °C with shaking at 150 rpm and cultivated as working stock. In order to perform the experiments, yeast cells were harvested by centrifugation at 3000× *g* for 10 min and then washed twice with phosphate-buffered saline (PBS, pH 7.0); finally, cell density was adjusted to 1 × 10^7^ cells/mL in SDB. 

#### 3.6.2. *C. albicans* Viability Analysis by Colony Forming Units (CFU)

CFU analysis was performed to test the effect of PMMA/AgNPs composite materials against *C. albicans*. PMMA resins, with AgNPs (3% and 3.5%) or without them as negative control, were incubated with 1 mL of yeast cell suspension (1 × 10^7^ cells/mL) for 24 and 48 h, then the Miles and Misra technique was employed [54]. Serial dilutions of *C. albicans* grown in presence of dental resins were plated on SDB agar Petri dishes and incubated at 37 °C for 24 h. Colonies were manually counted and the CFU was calculated using the following formula: CFU = Number of Colonies Counted/[Amount plated (in mL) × the dilution].

#### 3.6.3. *C. albicans* Adhesion Test

Initial adhesion and colonization of *C. albicans* on the surface of the samples were evaluated by adherence assays. The discs of dental materials with AgNPs (3.5 wt%) were placed in a 24-well tissue culture plate (Corning, St. Louis, MO, USA), and 1 mL of the yeast cell suspension (1 × 10^7^ cells/mL) was added. Resins without AgNPs represented negative control. The samples were incubated with *C. albicans* suspension at 37 °C for 24 and 48 h respectively. Then, dental materials were gently washed with sterile PBS to remove non-adherent cells, while remaining adherent cells were fixed by immersion in methanol (50% *v*/*v*) for 2 min and then left to dry.

#### 3.6.4. Analysis of *C. albican* Adhesion by Scanning Electron Microscopy (SEM) Acquisitions

After fixation, the samples were coated with gold (10 nm) and examined in a scanning electron microscope (SEM) JEOL JSM-6480LV (JEOL USA, Inc., Peabody, MA, USA) with a magnification up to 2000× operating at a maximum accelerating voltage of 20 kV. After SEM acquisitions, the yeast-free area in the captured images was manually traced and quantified using ImageJ public domain software (NIH) and its specific tools. Circularity values were obtained as means calculated on 20 *C. albicans* cells and statistically analyzed by means of a paired two-tailed *t*-test. 

Data reported were calculated as average ± SD on three independent experiments, and the values were considered statistically significant with respect to control for *p*-value ˂ 0.05 (<0.05 *, <0.01 ** and <0.005 ***).

## 4. Conclusions

Nowadays, PMMA represents suitable material for fabrication of full denture structures and removable devices, thanks to its intrinsic material properties and its high biocompatibility. Nevertheless, PMMA dental prostheses are subject to the colonization of *C. albicans*, inducing the development of serious oral infections. In this experimental work, we demonstrated how AgNP addition in acrylic resin matrix decreases the surface roughness, reducing the viability of *C. albicans* as consequence of a reduction of its ability to adhere and colonize PMMA dental prostheses. The results obtained open new perspectives in dentistry and they could be useful to increase the performance of the removable prosthesis by improving the patients’ quality of life by reducing oral infections.
